# Humoral and cellular immune durability of different COVID-19 vaccine platforms following homologous/heterologous boosters: one-year post vaccination

**DOI:** 10.3389/fimmu.2025.1526444

**Published:** 2025-01-22

**Authors:** Maaweya Awadalla, Halah Z. AlRawi, Rahaf A. Henawi, Fawziya Barnawi, Haitham Alkadi, Ahmed Alyami, Ammar Alsughayir, Alyazeed S. Alsaif, Ayman Mubarak, Wael Alturaiki, Bandar Alosaimi

**Affiliations:** ^1^ Research Center, King Fahad Medical City, Riyadh Second Health Cluster, Riyadh, Saudi Arabia; ^2^ Pathology and Clinical Laboratory Medicine Administration, King Fahad Medical City, Riyadh Second Health Cluster, Riyadh, Saudi Arabia; ^3^ Department of Botany and Microbiology, College of Science, King Saud University, Riyadh, Saudi Arabia; ^4^ Department of Medical Laboratory Sciences, College of Applied Medical Sciences, Majmaah University, Majmaah, Saudi Arabia

**Keywords:** humoral, cellular, durability, COVID-19, vaccine, homologous, heterologous, boosters

## Abstract

**Introduction:**

The durability of Hybrid immunity induced by natural infection and/or COVID-19 vaccines and evidence supporting further booster vaccination are crucial factors for pandemic response, yet remain poorly understood.

**Methods:**

We measured the durability of immune response and neutralizing capacity of antibodies following Homologous/Heterologous vaccination by mRNA-based vaccines (Pfizer-BioNTech BNT162b2) or (Moderna mRNA-1273) and viral vector-based vaccines (ChAdox1 nCoV-19-Oxford-AstraZeneca) in infected and non-infected patients. We also evaluated the long-lasting specific humoral IgG levels and T-cell immunity of the Memory CD8 cells.

**Results:**

We found that heterologous prime boosters led to significantly higher IgG antibody levels)9.09(than homologous boosters)5.236) one year after vaccination. We measured SARS-CoV-2 anti-S IgG antibodies and then assessed their neutralizing capacity to inhibit the receptor-binding domain (RBD) of the SARS-CoV-2 wild-type strain and omicron B.1.1.529/BA.2 variants from binding to the ACE2 receptors. The heterologous regiment demonstrated superior ACE2-binding inhibition and consistently had higher mean ACE2-receptor binding inhibition across all dose regimens without the need for further doses. The CD8+ T cells producing IFN-γ to various COVID-19 vaccine dose regimens were evaluated. We found that robust T cell mediated immune responses were preserved and largely induced by a heterogeneous vaccination eliciting a significantly higher CD8+ T cells IFN-γ response in 100% of vaccinees regardless of previous natural infection. Indeed, the difference between infected and naïve groups was less pronounced suggesting a reduced infection-related response.

**Discussion:**

Across three layers of evidence, this study showed that heterologous vaccination provides longer-lasting immunity than homologous doses, regardless of prior natural infection.

## Graphical Abstract

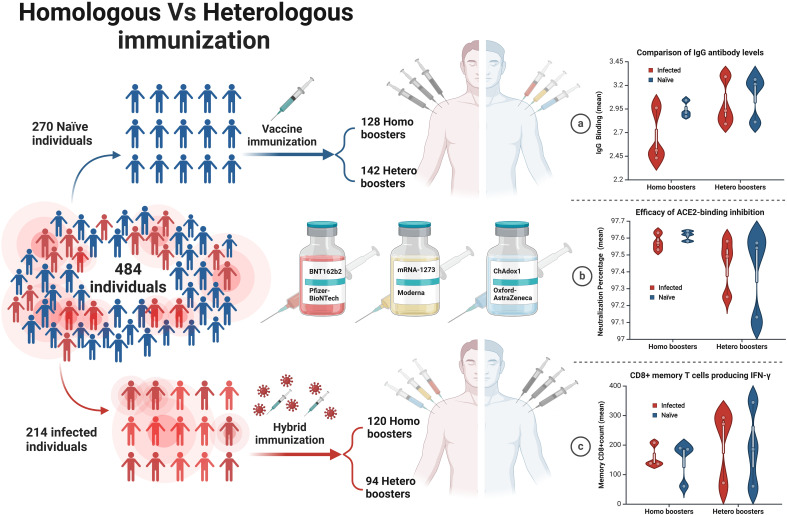

## 1 Introduction

During the COVID-19 pandemic, the response has relied largely on the development of vaccines. Investigating the durability of hybrid immunity induced by natural infection and/or COVID-19 vaccines is a crucial factor for the pandemic response. Several types of COVID-19 vaccines have received FDA approval to prevent COVID-19. Moderna Spikevax, Janssen (Johnson & Johnson), BioNTech Comirnaty, Pfizer/BioNTech Comirnaty, and Oxford/AstraZeneca Vaxzevria have all been approved for use in Saudi Arabia. In Phase 3 trials, a single dose of these vaccines proved remarkably effective with early vaccine efficacy of up to 95% against COVID-19 cases (1). However, the “real world” studies of vaccine effectiveness showed immune waning in the vaccinated population over time (2–4). Therefore, many countries worldwide have authorized the use of homologous and heterologous vaccine boosters (5). The durability of immunity and neutralizing capacity of antibodies following homologous and heterologous COVID-19 booster vaccinations remain poorly understood. Furthermore, the “hybrid immunity” or the immunity elicited against COVID-19 by vaccination and natural infection has not been fully considered in clinical trials efficacy nor real-world effectiveness studies.

The humoral and cellular immunity elicited by vaccines and infections are equally important when studying vaccine effectiveness. Although neutralizing antibodies have been measured as a correlate of protection against COVID-19 infection, accumulative evidence also considered T and B cell memory responses as key modulators in protective immunity (6). Unfortunately, with the concern of waning immunity suggested by studies relying on antibodies, many countries, including Saudi, introduced COVID-19 booster doses following the primary two doses of the immunization schedule. Although the homologous prime-boost strategy offered a reliable protective immunity against COVID-19, evidence for the efficacy of heterologous vaccination has been shown to significantly induce more immunogenicity across viral vector and mRNA vaccine platforms (7). Hence, our study was undertaken to determine the kinetics of vaccine-specific immune memory, assessing antibody, CD4+ T cell, CD8+ T cell, and memory B cell vaccine responses to different vaccine platforms.

SARS-CoV-2 has evolved rapidly and five major variants of concern were reported to the WHO within 2 years of the pandemic (8). The reliability of antibody levels as surrogate markers for immunity has created uncertainty regarding the robustness and duration of protection offered. Recent findings have shown that the Omicron predominantly became more transmissible and reduced the efficacy of vaccinations (9). In comparison to previous variants, Omicron exhibits a greater antigenic divergence from the ancestor virus. Consequently, the protective efficacy of previous vaccines against Omicron has significantly decreased (10). Because of the pandemic transmission of immune-evasive variants of concern, including omicron, many countries included a 3rd dose worldwide (11). The present study was designed to study the ACE2-binding inhibition against different Omicron Variants including B.1.1.529 and BA.2 in comparison to the wild type.

Understanding the long-term dynamics of SARS-CoV-2 antibodies and the duration of specific antibodies against omicron lineages in convalescent healthy individuals can provide information for improving vaccination strategies, understanding protective immunity against COVID-19 and assessing the likely course of future pandemics. Immunological memory of the dominant SARS-CoV-2 omicron lineages assessed for up to 12 months after vaccination helps to determine protection against reinfection, disease risk, and vaccine efficacy. Given the evidence that immunological memory can consist of memory B cells, antibodies, memory CD4+ T cells, and/or memory CD8+ T cells, that participate in protective immunity in the blood of vaccinated and infected subjects up to 12 months post-vaccination, we investigated interrelationships among multiple compartments of four types (antibody, memory CD4 and CD8 cells, IgG levels) of circulating immune memory to SARS-CoV-2. To the best of our knowledge, this is the first humoral and cellular evaluation of the homologous and heterologous kinetics of immune memory of vaccinated and infected subjects receiving the 2nd,3rd, and 4th boosters one-year post-vaccination.

## 2 Materials and methods

### 2.1 Study design and setting

Four hundred eighty-four healthy volunteers were recruited for our study. A consent form was signed during this process. Samples of blood were collected at a single time point. All participants were divided into three categories: two doses (either hetero or homo), three doses (either hetero or homo), and four doses (either hetero or homo). Plasma was separated by centrifugation from the participants and was taken in an anticoagulant tube. To prevent freeze-thaw cycling, the treated samples were divided into aliquots and stored at 80°C for further analysis.

### 2.2 Measurement anti-IgG SARS-CoV-2 using ELISA assay

A SARS-CoV-2 IgG ELISA kit (BGI Europe A/S) was used in accordance with the manufacturer’s instructions to identify SARS-CoV-2 anti-S IgG antibodies. IgG antibody ELISA kit has a 98.38% specificity and a 98.71% sensitivity, respectively. 96-well ELISA plates that had been pre-coated with pure SARS-CoV-2 viral antigen was used. In this experiment, positive and negative controls (to determine the cutoff) and blanks were utilized. The assigned wells received dilution-free additions of 100 microliters each of the positive and negative controls, while the blank well received no liquid additions at all. The plate will be incubated at 37°C for 30 min with 10 L of plasma, 20 L of saliva, and 100 microliters of sample diluent buffer added to each well. After that, an ELISA washer was used to wash the plates 3–5 times. Each well received a 100 L addition of anti-human IgG-HRP (conjugated antibody), which was then incubated for 20 min at 37°C. Each well received 50 L of substrates (A and B), which was then put to the plates and incubated for 10 min at 37°C in the dark after three rounds of washing. The stop solution was then added in 50 L portions to each well. At 450 nm, the optical density was calculated. In accordance with the kit’s instructions, the cutoff values (0.235) for anti-SARS-CoV-2 IgG antibody detection were calculated as follows: 0.1 + mean absorbance (0.135) of the two negative controls. The positive threshold of this test, as with most commercially available kits, is usually expressed in OD units.

### 2.3 PBMC isolation and *in vitro* expansion of SARS-specific T and B cells

PBMCs were isolated from fresh heparinized blood by density gradient centrifugation using Ficoll-Paque™ (GE Life Sciences) and resuspended in RPMI medium (Invitrogen) with 2% pooled bovine serum (10%AB). PBMCs was used directly for *in vitro* expansion in the presence of SARS-CoV-2 peptides. T-cells were cultured and stimulated overnight at 37 °C 5% CO_2_ in the presence of SARS peptide pools of PepTivator SARS-Cov-2 Port S complete (Catalogue No. 130-126-700), PepTivator^®^ SARS-CoV-2 Prot_S B.1.1.529/BA.5 WT Reference Pool (Catalogue No. 130-132-050), PepTivator^®^ SARS-CoV-2 Prot_S B.1.1.529/BA.5 Mutation Pool (Catalogue No. 130-132-051), PepTivator^®^ SARS-CoV-2 Prot_S B.1.1.7 Mutation Pool (Catalogue No. 130-127-844), PepTivator^®^ SARS-CoV-2 Prot_S B.1.1.529/BA.2 Mutation Pool (Catalogue No. 130-130-807), PepTivator^®^ SARS-CoV-2 Prot_S B.1.1.529/BA.1 Mutation Pool (Catalogue No. 130-129-928), PepTivator^®^ SARS-CoV-2 MHC-I Select (Catalogue No. 130-132-632) and PepTivator^®^ SARS-CoV-2 MHC-I Select Prot_S (Catalogue No. 130-130-633). All peptides purchased from Miltenyi Biotec as lyophilized powder. Cells were then washed twice with PBS and counted, then transferred to ELISPOT plate which is coated with anti-human IFN-γ, anti-human TNF and anti-human IgG.

### 2.4 SARS-CoV-2 (2019-nCoV) inhibition assay

Competitive ELISA was performed on Samples of SARS-CoV-2 vaccinated individuals. The SARS-CoV-2 (2019-nCoV) Inhibitor in the samples competes with ACE2-His to combine with immobilized SARS-CoV-2 S Protein RBD. The signal color becomes lighter as the content of the SARS-CoV-2 Inhibitor increases. We used the SARS-CoV-2 (2019-nCoV) Inhibitor Screening ELISA Kit (Sino Biological, Beijing, China) according to the manufacturer’s instructions. Plate was Pre-coated with SARS-CoV-2 (2019-nCoV) Spike RBD-mFc Recombinant Protein, SARS-CoV-2 Inhibitor was used as control and Human ACE2 (His Tag) recombinant protein was used (all provided with the kit. Washing was performed using Wellwash™ Versa Microplate Washer (Thermo Scientific, Waltham, MA USA), and optical density (O.D.) was read at 450 nm using Multiskan FC Microplate Photometer (Thermo Scientific, Waltham, MA USA). The positive and negative critical values of the kit can be used to judge whether the sample has a neutralization effect. Inhibition was quantified using the following equation:


$$Inhibition=1-\frac{\text{OD value of Sample}}{\text{OD value of Negative Control}}\;x\;100\text{ \%}$$

### 2.5 Anti-human ELISpot assays

Two T-cell and one B-cell ELISpot Kits (U-CyTech biosciences, Netherlands) were used according to the manufacturer’s instructions. In brief, T-cell plates were coated with anti-human Interferons gamma IFNγ and anti-tumor necrosis factor TNF, where B-cells were coated and anti-human IgG antibody and incubated overnight at 4 °C. 300,000 PBMCs were cultured per well and stimulated *in vitro* for 18 h with pools of SARS-CoV-2 peptides. Peptide pools (1-2 μg/ml) were used. After 18 hours of incubation, the ELISpot plates were treated with their corresponding human biotinylated detection antibody, followed by incubation with streptavidin-AP and KPL BCIP/NBT Phosphatase Substrate. Spot forming units (SFU) were quantified with ImmunoSpot. To quantify positive T and B cells-specific cell-specific responses, spots of the unstimulated cells were subtracted from the peptide-stimulated cells, and the results were counted and expressed as SFU/300,000 PBMCs.

### 2.6 Statistical analysis procedure

All categorical variables such as gender, age group, infection, immunity, and vaccine status, and presented as frequencies and percentages. Continuous variables such as age, the time difference between vaccination and infection, IgG antibody (unit/mL) are expressed as Mean ± SD. The Kolmogorov-Smirnov test was used to confirm the assumption of normal distribution. If the data was biased, a nonparametric test was used. Pearson chi-square/Fisher’s exact test was used to determine significant associations between categorical variables, depending on whether the cell was expected to have an expected frequency of less than 5. The independent sample t-test was applied to determine the mean significant difference between IgG level and vaccine status, IgG level among infected patients. Box plot was used to identify the difference in IgG level among age groups, gender, vaccine status, and infected patients. Pearson’s correlation coefficient test was applied to determine a significant relationship between age and IgG level. A two-sided p-value less than 0.05 was considered statistically significant. All data was entered and analyzed using the SPSS 25 Statistics Package (SPSS Inc., Chicago, Illinois, USA) and MEDCALC version 18.11.6 (Acacialaan 22 8400 Ostend Belgium).

### 2.7 Ethical approval

Ethical approval for the study was obtained from the Institutional Review Board (IRB) committee at King Fahad Medical City (KFMC) under reference number 22-501. All work was conducted according to the principles of the Declaration of Helsinki. All participants provided written and signed informed consent.

## 3 Results

### 3.1 COVID-19 vaccine cohorts

To compare the development of immune memory, we enrolled a total of 484 participants who received immunization with mRNA-based vaccines (Pfizer-BioNTech BNT162b2) or (Moderna mRNA-1273) and viral vector-based (Oxford/AstraZeneca ChAdOx1) vaccines. Information on the history of vaccination and previous SARS-CoV-2 infection was retrieved from the Tawakkalna mobile application which is the official App used to verify vaccination status, current or previous infection, and contact tracing in Saudi Arabia. Stratification of infection status was confirmed by IgG measurement against the Nucleocapsid (N) protein in collected samples. Both cohorts of 214 infected and 270 non-infected received a minimum of two doses of homologous or heterologous vaccine, approximately 28 and 21 days apart. Blood samples were collected at one-time points between Dec 2022 and May 2023, and both plasma and peripheral blood mononuclear cells (PBMC) were preserved. The mean age of the participants was 32.6 ± 9.5 and 35.3 ± 10 years for the infected and non-infected groups, respectively. Males represented 80% and females 20% of the total cohort. All vaccine groups were similar in their distribution of gender, nationality, and reported comorbidities. Characteristics of the participants’ cohorts are shown in **Table 1**.

**Table 1:** Master table.

Vaccine regiments		Infected (214)		Non-infected (270)		
		Homologous	Heterologous	Homologous	Heterologous	P-value
Number of vaccinees		120	94	128	142	0.83
Age groups	< 40	89	75	91	93	0.58
	> 40	31	19	37	49	0.99
Nationality	Saudi	105	55	78	51	0.11
	Non-Saudi	15	39	50	91	0.41
Gender	Male	96	61	101	129	0.93
	Female	24	33	27	13	0.82
Chronic diseases	Yes	9	26	7	5	0.55
	NO	111	68	121	137	0.51

### 3.2 Comparison of IgG antibody levels between different homologous and heterologous COVID-19 vaccine platforms

**Figure 1:**
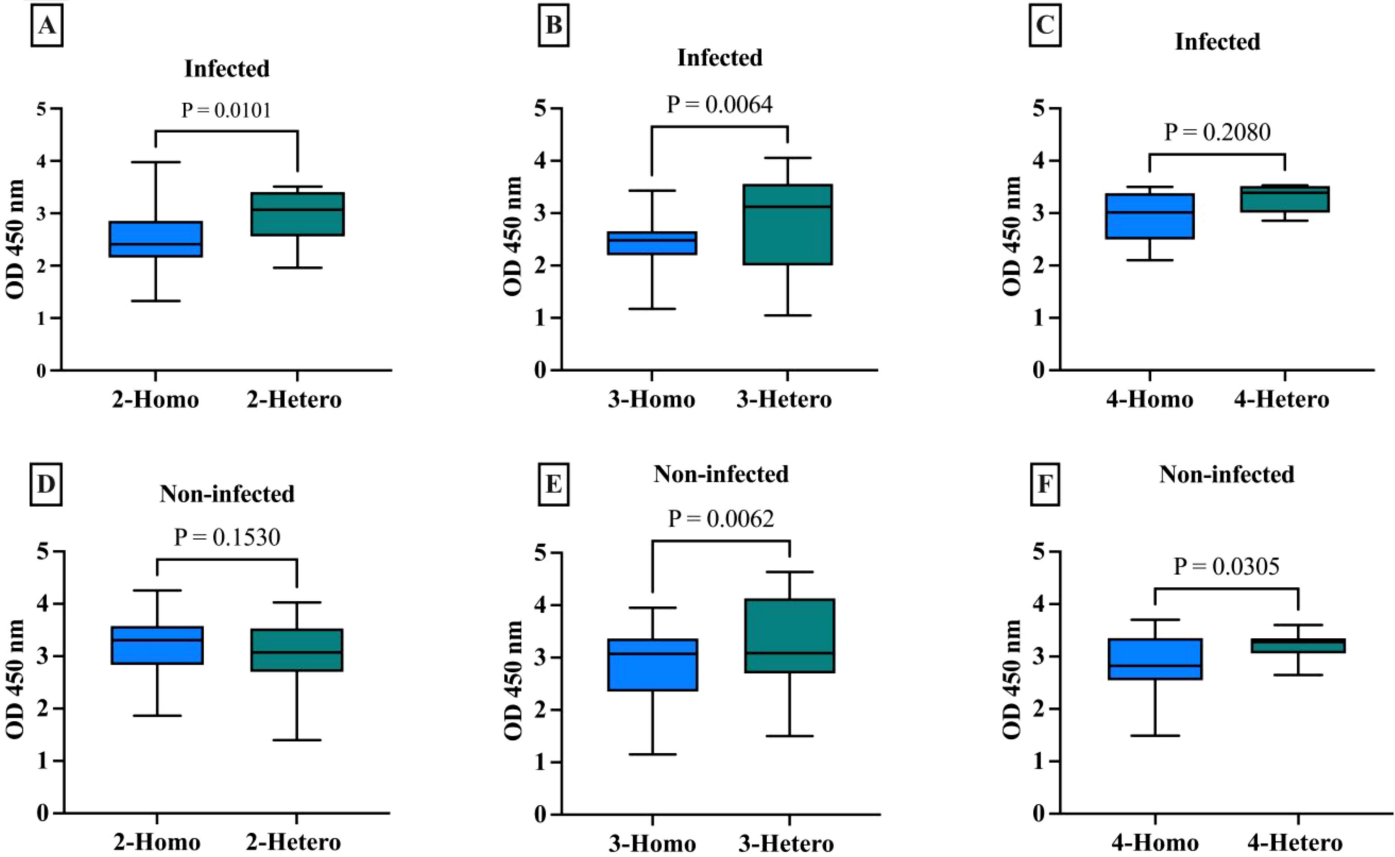
Comparison of IgG antibody levels with different numbers of doses across homologues and heterologous vaccines in infected and non-infected groups. **(A)** 2-dose homologous and heterologous COVID-19 infected group. **(B)** 3-dose homologous and heterologous COVID-19 infected group. **(C)** 4-dose homologous and heterologous COVID-19 infected group. **(D)** 2-dose homologous and heterologous COVID-19 non-infected group. **(E)** 3-dose homologous and heterologous COVID-19 non-infected group. **(F)** 4-dose homologous and heterologous COVID-19 non-infected group. The box plots show the middle line representing the median, the box representing the interquartile range, and the whiskers extending to the minimum and maximum values. The p-values indicate the statistical significance of the differences between the homologues and heterologous groups for each dose of the COVID-19 vaccine group.

The antibody response was compared between the different vaccine doses whether homologous or heterologous in the infected and non-infected groups. For the 2-dose regimens in infected individuals, the heterologous formulations had a mean IgG level of 2.93, which was 0.42 points higher than the 2.51 mean for the homologous mRNA group (p=0.0101), indicating the 2-dose heterologous regimen elicited a stronger IgG antibody response compared to the 2 doses homologous mRNA regimen (**Figure 1A**). The difference was even larger in the 3-dose groups, where the heterologous formulations had a mean IgG of 2.78, 0.36 points higher than the 2.43 mean for the homologous mRNA group (p=0.0064) (**Figure 1B**), demonstrating the clear superiority of the 3-dose heterologous approach over the 3-dose homologous mRNA regimen in inducing IgG antibodies. For the 4-dose regimens, the heterologous group had a mean IgG of 3.38, 0.42 points above compared to 2.96 for the homologous mRNA group (p=0.2080) (**Figure 1C**), suggesting the 4-dose heterologous formulation also generated a stronger IgG response than the 4-dose homologous mRNA, although not statistically significant.

The 2-dose heterologous regimen in non-infected individuals (mean 3.21), showed no statistically significant difference (p=0.1530) between IgG antibody response and the homologous mRNA group (mean 3.05) (**Figure 1D**). This difference was larger in the 3-dose groups, with the heterologous regimen showing clear superiority (p=0.0062) (**Figure 1E**). The 4-dose heterologous group also generated a stronger IgG response than the homologous mRNA group (p=0.0305) (**Figure 1F**).

### 3.3 Correlation between the IgG levels and the ages within each vaccine group

To further elucidate the data, we analyzed any potential patterns or relationships between the IgG levels and the ages of participants within each vaccine group. This analysis aimed to determine if there were differences in IgG levels between younger and older people who received the same vaccine.

**Figure 2:**
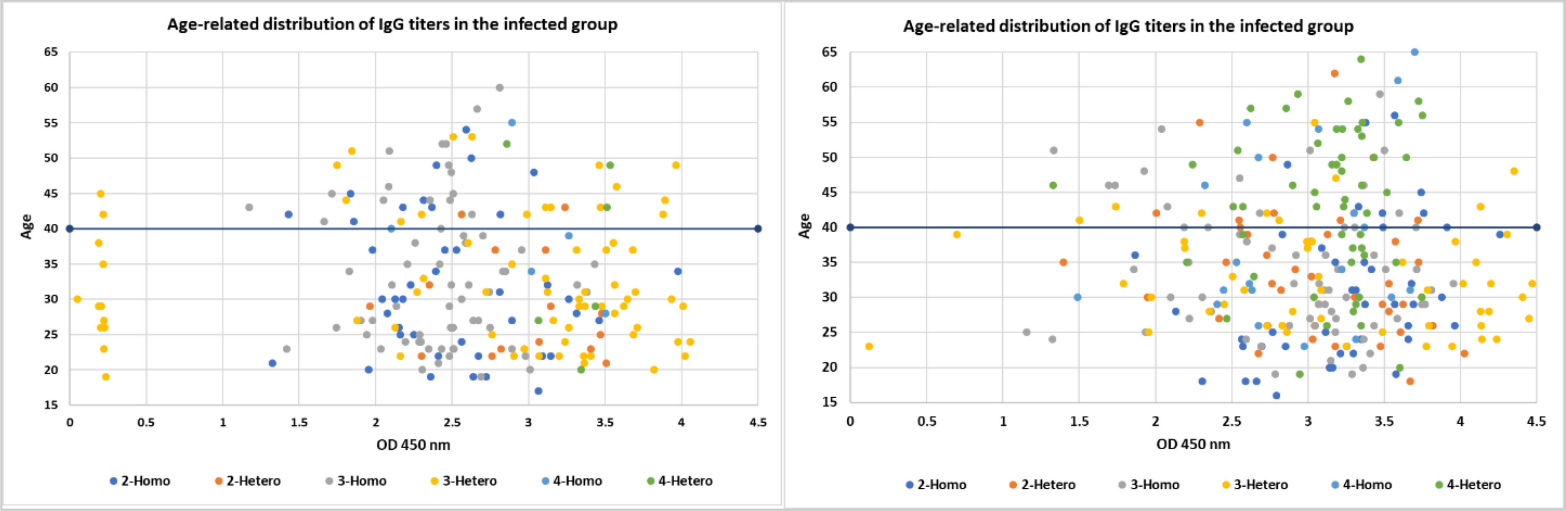
The figure consists of scatter plots comparing the age-related distribution of IgG titers within the infected and non-infected groups. Each color represents a different COVID-19 vaccine dose defined by heterologous or homologous regiment and the respective IgG levels. The x-axis likely represents age and the y-axis represents the IgG levels.

Among the infected group (**Figure 2**), the highest IgG values were fairly dispersed across all ages with a clear pattern observed in the younger age <40 years old. The lower IgG levels were found to be among the 3-dose heterologous individuals. The 2-dose vaccinated individuals exhibited a clear relationship between IgG levels in the younger ages, with only 2 individuals who received 2-hetero doses appearing in the older age group of >40 years old. This suggests younger individuals may mount a more robust humoral immune response to a 2-dose vaccine than older adults. This trend indicates that age may be a major factor influencing the humoral immune response to a 2-dose heterologous vaccine regimen. This also suggests that, if infected, a heterologous dose of any 2 vaccine regimens may be effective in generating a robust humoral response regardless of age.

Similarly, the non-infected group exhibited a correlation between IgG levels and age with younger individuals (<40) mounting a more robust humoral immune response to vaccine regimens than older adults (>40). Although a clear age-related pattern was observed, the IgG values did not show any variation or outlier values.

Comparing the two groups, the correlation between IgG levels and age was pronounced in the younger individuals (<40) compared to the older adults (>40) group. Even without a prior infection, the lowest number of doses was sufficient to induce a robust humoral response. These findings provide insights into the potential of infection and age-related differences in humoral immune responses to various COVID-19 vaccine regimens, which may inform vaccination strategies and guidance, particularly in the context of optimizing vaccine-induced immunity across different age groups.

### 3.4 Comparative efficacy of ACE2-binding inhibition against the wild-type and omicron variants across homologous and heterologous COVID-19 vaccine regimens

**Figure 3:**
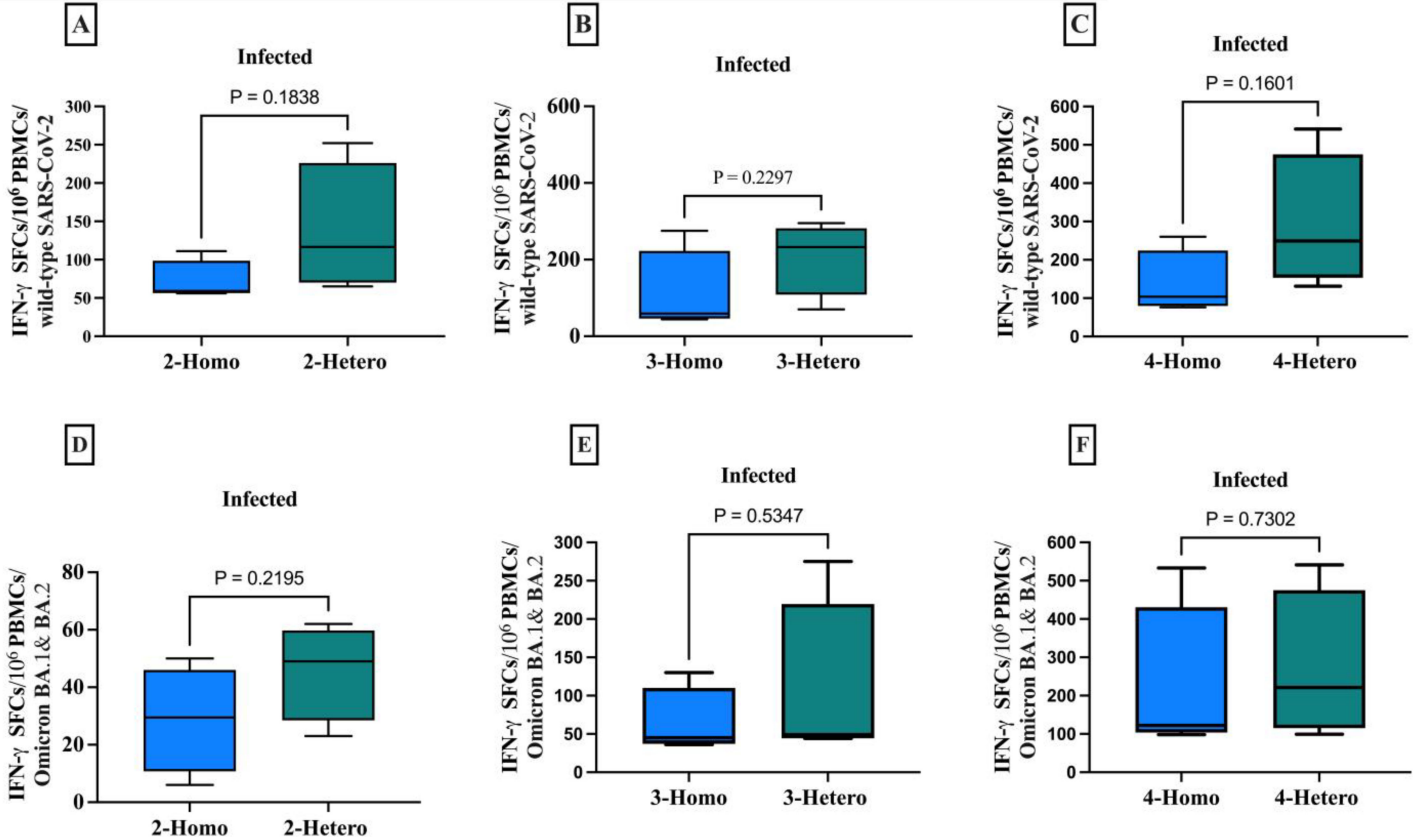
The CD8+ memory T cells producing IFN-γ against the SARS-CoV-2 wild-type and Omicron B.1.1.529/BA.2 variants among infected individuals. The comparison between homologous and heterologous vaccine regimens is presented as the number of IFN-γ secreting CD8+ T cells against **(A)** 2-doses wild-type **(B)** 3-doses wild-type **(C)** 4-doses wild-type **(D)** 2-doses Omicron B.1.1.529/BA.2 variants **(E)** 3-doses Omicron B.1.1.529/BA.2 variants **(F)** 4-doses Omicron B.1.1.529/BA.2 variants. PBMCs were re-stimulated with wild-type SARS-CoV-2 and Omicron B.1.1.529 and BA.2 variants peptide pools and cultured in a 96-well ELISpot plate for 20 hours. This allowed the detection of CD8+ T cells producing IFN-γ, measured as Spot-forming cells (SFCs) using IFN-γ ELIspots. The box plots show the middle line representing the median, the box representing the interquartile range, and the whiskers extending to the minimum and maximum values. The p-values indicate the statistical significance of the differences between different doses of the COVID-19 vaccine dose group.

To evaluate the functionality of COVID-19 vaccine-induced IgG antibodies, we assessed their ability to inhibit the virus receptor-binding domain (RBD) binding to the ACE2 receptor (**Figure 3**) (**Table 2**). This is a surrogate measure of neutralizing capacity against the SARS-CoV-2 wild-type strain and omicron B.1.1.529/BA.2 variants using one-way ANOVA tests. The binding rates of IgG antibodies from the wild-type were not statistically significant between homologous and heterologous vaccines for wild-type SARS-CoV-2, except in the 3-dose group (p = 0.0411) among the infected group.

**Table 2:** ACE2-binding inhibition against various SARS-CoV-2 variants by different doses of heterologous or homologous regiment and COVID-19 infection status.

Infected									
Type- Number of doses	Homo-2	Hetero-2	P-value	Homo-3	Hetero-3	P-value	Homo-4	Hetero-4	P-value
	MI %	MI %		MI %	MI %		MI %	MI %	
Wild type	97.58%	97.53%	0.12	97.49%	97.57%	0.04	97. 25%	97.63%	0.81
Omicron Variant B.1.1.529	66.75%	75.09%	0.03	62.45%	80.17%	0.03	67.81%	88.91%	0.38
Omicron BA.2 Variant	76.04%	83.82%	0.42	82.04%	90.84%	0.15	88.93%	92.86%	0.20

Non-Infected									
Type- Number of doses	Homo-2	Hetero-2	P-value	Homo-3	Hetero-3	P-value	Homo-4	Hetero-4	P-value
	MI %	MI %		MI %	MI %		MI %	MI %	
Wild type	97.13%	97.58%	0.41	97.57%	97.62%	0.55	97.54%	97.64%	0.3
Omicron Variant B.1.1.529	69.28%	78.65%	0.35	74.88%	87.50%	0.785	86.56%	90.93%	0.47
Omicron BA.2 Variant	85.61%	86.32%	0.90	83.09%	94.15%	0.0001	84.30%	96.24%	<0.0001

*MI %, Percentage of Mean Inhibition.

When we compare the ACE2-receptor binding inhibition between the homologous mRNA and heterologous groups against the omicron variant B.1.1.529, there were statistically significant differences between the two groups (p=0.03) for both the two-dose and three-dose regimens. However, there was no statistically significant difference in the ACE2-receptor binding inhibition between the homologous and heterologous groups against the rest of omicron variant B.1.1.529 in the non-infected group.

Similarly, ACE2-binding inhibition to the omicron variant BA.2 among infected individuals across all dose regimens (2-dose, 3-dose, and 4-dose) among infected individuals was not statistically significant. However, in the three-dose vaccination group, the heterologous regimen displayed significantly higher ACE2-binding inhibition (mean 94.15%) compared to the homologous regimen (mean = 83.09%, p = 0.0001). Similarly, in the four-dose group, the heterologous regimen (mean 96.24%) showed significantly higher inhibition compared to the homologous regimen (mean 84.30%, p=<0.0001). This suggests that the 3-dose heterologous regimen was sufficient for the non-infected individuals, eliminating the need for a 4-dose heterologous regimen, thereby reducing potential costs associated with additional vaccination.

### 3.5 Vaccine-induced T cell immune responses against wild-type SARS-CoV-2 and Omicron variants

**Figure 4:**
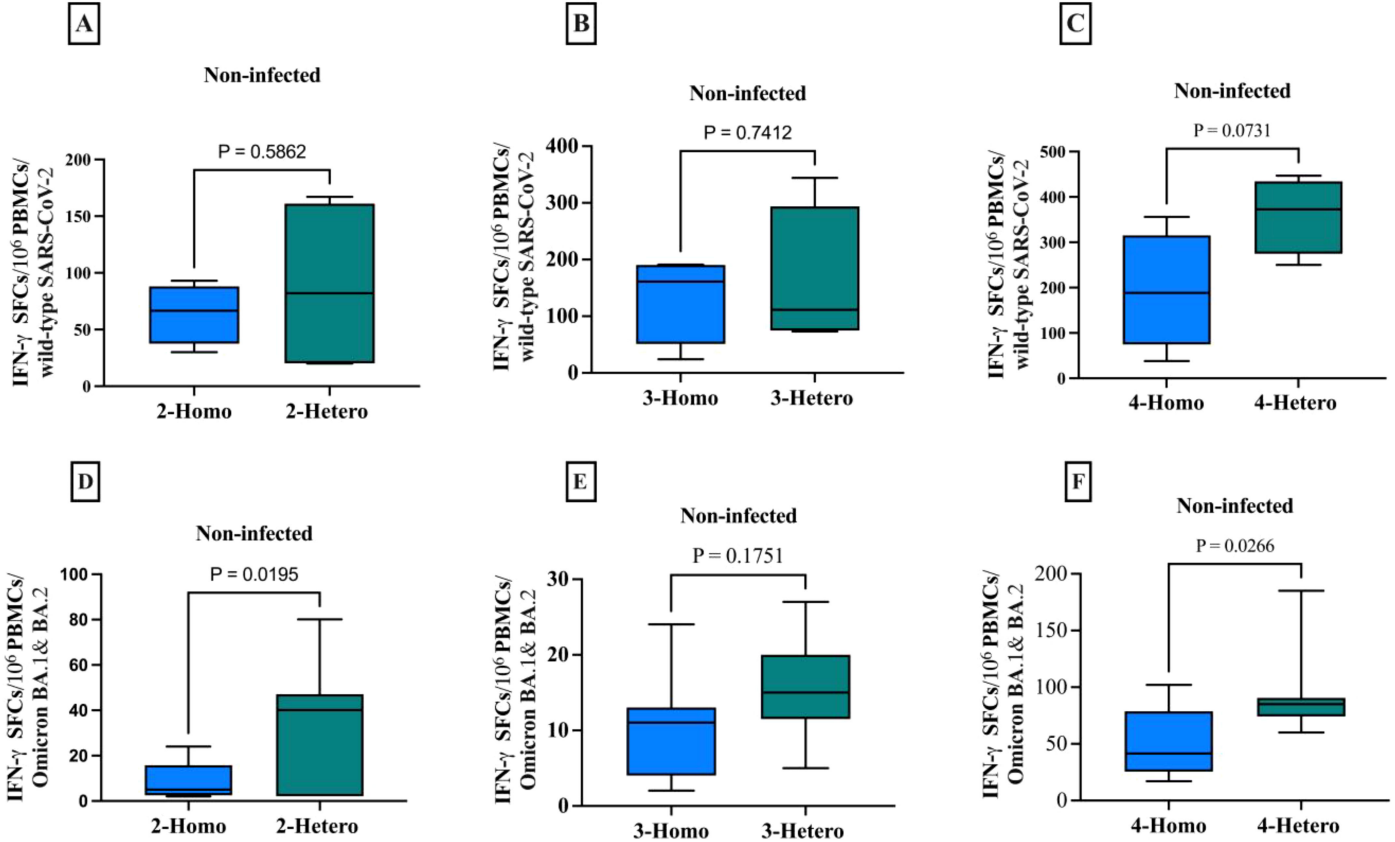
The CD8+ memory T cells producing IFN-γ against the SARS-CoV-2 wild-type and Omicron B.1.1.529/BA.2 variants among non-infected individuals. The comparison between homologous and heterologous vaccine regimens is presented as the number of IFN-γ secreting CD8+ T cells against **(A)** 2-doses wild-type **(B)** 3-doses wild-type **(C)** 4-doses wild-type **(D)** 2-doses Omicron B.1.1.529/BA.2 variants **(E)** 3-doses Omicron B.1.1.529/BA.2 variants **(F)** 4-doses Omicron B.1.1.529/BA.2 variants. PBMCs were re-stimulated with wild-type SARS-CoV-2 and Omicron B.1.1.529 and BA.2 variants peptide pools and cultured in a 96-well ELISpot plate for 20 hours. This allowed the detection of CD8+ T cells producing IFN-γ, measured as Spot-forming cells (SFCs) using IFN-γ ELIspots. The box plots show the middle line representing the median, the box representing the interquartile range, and the whiskers extending to the minimum and maximum values. The p-values indicate the statistical significance of the differences between different doses of the COVID-19 vaccine dose group.

CD8+ T cells producing IFN-γ to various COVID-19 vaccine dose regimens were evaluated in infected individuals against both the wild-type SARS-CoV-2 and Omicron B.1.1.529 and BA.2 variants. Although not statistically significant, heterologous regimens had a higher average of CD8+ T cells producing IFN-γ per 1x106 PBMCs than homologous regimens, when tested against the peptide pools of the ancestral wild-type SARS-CoV-2 and Omicron B.1.1.529/BA.2 variants (**Figure 4**). Although not statistically significant, the heterologous vaccines had a markedly higher mean of CD8+ T cells producing IFN-γ count against both the wild-type SARS-CoV-2 and Omicron B.1.1.529/BA.2 variants across all doses of vaccine regimens. Which means the 2-doses elicited sufficient memory T cell immunity, eliminating the need for further vaccinations.

In non-infected individuals, the heterologous regimen elicited a significantly higher CD8+ T cells IFN-γ response (p=0.0195) and (p=0.0266) compared to the homologous regimens against Omicron B.1.1.529 and BA.2 variants particularly, in comparison to the wild-type.

## 4 Discussion

Little is known about the difference between mRNA-primed and viral-vector-primed individuals in terms of humoral and cellular immune response and the impact of infection up to 1 year after homologous and heterologous vaccination. Here, we found that heterologous prime boosters led to significantly higher IgG antibody levels than homologous boosters one year after vaccination. The heterologous approach appears to be the superior strategy for generating a potent humoral immune response against SARS-CoV-2. The overall trend of COVID-19 vaccine IgG levels suggests that with 2-doses only, the infected group has significantly higher IgG levels compared to the non-infected group, indicating a potential infection-related response. As the dose increases to 4, the difference in IgG levels between infected and non-infected groups becomes less pronounced, potentially suggesting a reduced infection-related response at higher doses. These finding are in agreement with several studies demonstrated that protection conferred by natural infection was projected to wane (12–14). The decline of anti-SARSCoV-2 IgG levels over one-year post vaccination is in line with other reports (15, 16). In this study, the clinical significance of our approach predicted that heterologous vaccination would result in a renewed durability that endures for a longer time than the period of immunity conferred by homologous doses.

Our study demonstrated the presence of long-term immune memory one-year post-vaccination. The heterologous two-dose regimen elicited robust memory T cell immunity, thereby eliminating the need for additional doses. Moreover, this heterologous approach exhibited superior ACE2-binding inhibition, consistently showing higher mean ACE2-receptor binding inhibition across all dosing regimens without requiring further doses. Interestingly, other research has indicated that months after the second dose of mRNA vaccination, 86% of immunized subjects retained a population of Spike-specific B lymphocytes, a significantly higher frequency compared to non-immunized controls. Additionally, specific CD4+ and CD8+ T lymphocytes capable of responding *in vitro* to stimulation with Spike-derived peptides were also present. These findings support the conclusion that vaccination with BNT162b2 induces a specific and potentially long-lasting immune response (17). However, some studies suggest that the immune response generated by mRNA vaccines may wane over time, leading to reduced protection against infection. For example, research indicates that antibody levels decline significantly within months following the second dose, particularly among older adults and immunocompromised individuals (18). This decline raises concerns regarding the duration of immunity and the risk of breakthrough infections. The ongoing scientific discussion about the necessity for booster doses has important implications for public perception and vaccine hesitancy. Some individuals may interpret the need for additional doses as evidence of the initial vaccination’s ineffectiveness, despite substantial evidence supporting the overall benefits of mRNA vaccines in reducing severe disease and hospitalization (19).

A study by WY and colleagues in 2022 reported a contrasting immune response pattern in support of homologous mRNA booster vaccination and reporting a modest effectiveness of heterologous booster vaccination even against the Omicron B.1.1.529 variant (20). Our results should be interpreted with consideration of the effect of the emergence of escape variants. To evaluate the functionality of COVID-19 vaccine-induced IgG antibodies, we assessed their neutralizing capacity to inhibit the receptor-binding domain (RBD) of the SARS-CoV-2 wild-type strain and omicron B.1.1.529/BA.2 variants from binding to the ACE2 receptors. Interestingly, a higher homologous vaccine dosing did not enhance ACE2-binding inhibition indicating that the heterologous prime-boost seemed to matter. The heterologous regiment demonstrated superior ACE2-binding inhibition and consistently had higher mean ACE2-receptor binding inhibition than the corresponding homologous groups across all dose regimens. These findings support the use of heterologous prime-boost vaccine regimen schedules to optimize protection against the wild-type and Omicron variants. This is a significant finding, as a 2-dose heterologous prime-boost vaccine was sufficient to achieve a high level of ACE2-binding inhibition compared to a 3-dose homologous regimen without the need for a fourth dose. This is supported by Hyun et al. in 2023 who found that the cross-reactive neutralizing activities against Omicron subvariants were negligibly low in the homologously primed population; however, at one-month post heterologous booster vaccination, the neutralizing activities against Omicron subvariants were enhanced (21).

The overall mean inhibition was similar in infected and non-infected groups across all neutralized variants indicating that immunity conveyed by vaccines is likely to induce immunogenicity and last for the same period as the immunity from natural infection. This could be explained by the fact that vaccines that target antigenic genes or conserved regions confer greater durability of immunity against the wild type and are likely to last longer than immunity from natural infection (8, 22). In the present study, ACE2-binding inhibition against Omicron subvariants have shown low cross-reactive neutralization activity. Recent studies have also found low neutralization between Omicron subvariants because of the spike RBD mutations of L452R, F486V, and R493Q of Omicron BA.5 (23) as well as the L452Q, L452R, and F486V mutations in the spike RBD of Omicron BA.4 (24–26). This demonstrates that the durability of neutralization efficacy is affected by the continuous evolution of SARS-CoV-2 variants during this pandemic enabling emergent sub-variants to overcome immunological memory.

Vaccine effectiveness has largely relied on the humoral arm of immunity despite the fact that spike-specific T-cells are the most strongly induced by vaccination (27). Understanding humoral as well as cellular immune responses against Omicron variants elicited by vaccination would be helpful for the design of more effective vaccines. Depending on the route of vaccination technology, the humoral vaccine-elicited neutralizing immunity has reduced to various degrees over a short period of time (8). However, there is compelling evidence that robust T cell-mediated immune responses are largely preserved, which explains reduced clinical severity and death despite high infectivity and transmissibility rates. CD8+ T cells producing IFN-γ to various COVID-19 vaccine dose regimens were evaluated in infected individuals against both the wild-type SARS-CoV-2 and Omicron B.1.1.529 and BA.2 variants. We found that the heterologous regimen elicited a significantly higher CD8+ T cells IFN-γ response compared to the homologous regimens. Our findings are supported by previous reports that stronger cellular immunity was induced by a heterogeneous mRNA-ChAd vaccination (27). Richardson and colleagues also reported superior cellular immunity by heterologous vaccination, reporting high IFN-γ release in the heterogeneous group who received the adenoviral vector-based (ChAdOx1) and mRNA Pfizer-BioNTech (BNT162b2) vaccines (28). This phenomenon could be attributed to the fact that the kinetics of adenovirus vector-based vaccines elicited a durable and strong CD8 T-cell response following vaccination (29). Interestingly, spike-specific memory CD8+ T cell responses were detected in 100% of vaccines regardless of previous natural infection. As expected, infected individuals generated circulating spike-specific CD8+ T cell memory frequencies similar to or higher than non-infected individuals at one-year post-vaccination. However, the analysis reported herein further expands previous findings, including three different vaccines and boosters representing two vaccine platforms, with evaluation recognition of variants, and consideration of previous natural infection at humoral and cellular levels (30).

Our primary focus on CD8+ T cell responses stems from their critical role in directly eliminating virus-infected cells and providing robust protective immunity. Moreover, memory CD8+ T cells are essential for protection against secondary infections, making them a key area of interest in immunological research and vaccine development. Previous studies have highlighted the importance of CD8+ T cells, particularly in the context of viral infections. By assessing CD8+ T cell responses, we aimed to enhance our understanding of the overall immune landscape. Future study should encompass a more comprehensive evaluation, including B-cell and CD4+ T cell responses, to provide a fuller picture of the immune memory established by vaccination.

Our study has several limitations. First, this study did not include participants vaccinated with the homogeneous viral vector vaccine. Second, we measured only CD8 T-cell response but other immunological factors such as B-cell and CD^4^ T-cell memory were not evaluated. Third, in this study we did not include adenovector vaccine controls. We believe that including these controls in future studies would provide a more comprehensive understanding of the immune responses elicited by various vaccine platforms. Due to the mass vaccination strategy in Saudi Arabia, we found a limited number of one-dose vaccinees, therefore, based the analysis on two, three, and four doses.

## 5 Conclusions

At one-year post-vaccination, the heterologous prime boosters induced significantly higher IgG antibody levels generating a potent humoral immune response against SARS-CoV-2 compared to their homologous counterparts. Furthermore, the heterologous regiment demonstrated superior ACE2-binding inhibition and consistently had higher mean ACE2-receptor binding inhibition across all dose regimens without the need for further doses. We found that robust T cell-mediated immune responses were preserved and largely induced by a heterogeneous vaccination eliciting a significantly higher CD8+ T cells IFN-γ response in 100% of vaccinees regardless of previous natural infection. Indeed, the difference between infected and naïve groups was less pronounced suggesting a reduced infection-related response. Overall, across three layers of evidence, this study showed that heterologous vaccination provides longer-lasting immunity than homologous doses, regardless of prior natural infection.

## Data Availability

The raw data supporting the conclusions of this article will be made available by the authors, without undue reservation.
